# Salivary Inflammatory Molecules as Biomarkers of Sleep Alterations: A Scoping Review

**DOI:** 10.3390/diagnostics11020278

**Published:** 2021-02-10

**Authors:** Vanessa Ibáñez-del Valle, Rut Navarro-Martínez, Maria Luisa Ballestar-Tarín, Omar Cauli

**Affiliations:** 1 Department of Nursing, Faculty of Nursing and Podiatry, University of Valencia, c/Jaume Roig s/n, 46010 Valencia, Spain; vanessaidelvalle@gmail.com (V.I.-d.V.); Rut.Navarro@uv.es (R.N.-M.); M.luisa.ballestar@uv.es (M.L.B.-T.); 2 Frailty and Cognitive Impairment Organized Group (FROG), University of Valencia, 46010 Valencia, Spain; 3 Chair of Active Ageing, University of Valencia, 4610 Valencia, Spain

**Keywords:** cytokine, saliva, sleep, inflammation, biomarkers

## Abstract

Poor sleep quality and sleep disorders are the most common problems in people, affecting health-related quality of life. Various studies show an association between sleep disorders and altered levels of stress hormones and inflammatory cytokines measured in saliva. The main objective of this article is to provide an analysis of the current evidence related to changes in inflammatory markers in the saliva and their associations with sleep quality measurement (both objective and subjective methods) in healthy subjects and in sleep-related disorders. To that end, a scoping review was carried out, following the PRISMA criteria in the bibliographic search in several databases: PubMed, EBSCO, and SCOPUS. Eleven of the articles are from the adult population and two from the child-youth population. They mainly measure the relationship between sleep and interleukin-1β (IL-1β), interleukin-6 (IL-6), and tumor necrosis factor alpha (TNFα) alpha, as well as other inflammatory markers such as myeloperoxidase (MPO) and prostaglandin-endoperoxide synthase 2. An analysis shows the relationship between these salivary biomarkers and sleep quality, especially in the case of IL-6 in both healthy subjects and several pathologies associated with sleep-disorders. The results for TNFα and IL-1β measurements are still inconclusive and the difference with IL-6 was assessed. Two studies reported interventions that result in sleep improvement and are accompanied by the normalization of inflammatory changes detected in the saliva. As it is an easy-to-apply and non-invasive method, the measurement of salivary cytokines can be very useful in chronobiology studies. Further studies are required to determine the sensitivity of salivary inflammatory markers in monitoring biological rhythms and acting as biomarkers in the detection of sleep disorders and sleep interventions.

## 1. Introduction

Sleep is a vital physiological process with important restorative functions [1]. However, poor quality of sleep is a common problem, to the extent that according to various studies, between 37.2% and 69.4% of adults sleep poorly [2]. 

Chronic sleep deprivation and sleep fragmentation are prevalent conditions in modern lifestyles and in several clinical settings [3,4,5,6]. Disorders of sleep fragmentation including sleep apnea, periodic limb movements, and other causes, such as pain and medications also greatly impair the quality of life and contribute to the worsening of pre-existing diseases [7,8,9]. Among psychological consequences, sleep disorders produce daytime sleepiness, decreased psychomotor performance, and degraded mood [10,11,12] and several studies demonstrated that sleep disorders also promote cardiovascular and metabolic disorders [5,13].

For this reason, sleep problems are a growing concern for global public health, since shorter sleep or sleep fragmentation have been shown to negatively influence the risk of inflammatory disease and to contribute to mortality [14], possibly due to the effects of sleep disturbance on inflammatory mechanisms. The release of GH and prolactin takes place during night-time sleep, and particularly the deep sleep characterized by slow wave sleep (SWS) that occurs during the during the first half of sleep. This endocrine environment during early sleep supports the production of pro-inflammatory and/or Th1 cytokines such as interleukin (IL) -1, IL-12, tumor necrosis factor (TNFα), and interferon (IFN) -γ [15]. In contrast, the activity of the two main stress axes, the hypothalamic pituitary adrenal axis (HPA) and the sympathetic nervous system (SNS) are suppressed during this sleep phase, and the anti-inflammatory actions of their respective effector hormones, cortisol and catecholamines (epinephrine and norepinephrine), are at their lowest levels [16]. and unlike short periods of sleep deprivation, they are conditions of profound stress that result in deleterious changes in the immune function [15]. In this context, numerous experiments have shown a consistent pattern of endocrine and immune rhythms which reflect a pro-inflammatory state during nighttime sleep, while wakefulness associated with rise in cortisol and catecholamines concentration is associated with anti-inflammatory effects [15,17]. However, the interplay between sleep and inflammation is complicated and it is not fully elucidated since under pathological conditions associated with sleep disorders or sleep alterations induced by work shifts, the prolonged loss of sleep or sleep fragmentation is accompanied by an increased release of pro-inflammatory substances [18,19,20].

Reduced hours of sleep therefore increase the secretion in blood of C-reactive protein (CRP), IL-6 and TNF-α [18,19,20]. Some of these cytokines, in turn, activate the transcription factor NF-κB, which acts as a mediator of inflammatory responses [21]. Unlike the pro-inflammatory state induced during SWS, the increase in pro-inflammatory activity after persistent sleep deprivation, although generally small given its persistent nature, can therefore be regarded as a non-specific state of chronic stress [15]. This state of low-grade chronic systemic inflammation is associated with a wide variety of diseases, including diabetes [22], cardiovascular disease [23], certain forms of chronic pain [24], certain cancers [25], and neurodegenerative diseases [26]. Likewise, sleep disturbances are present in all these diseases [27]. 

The fact that some cytokines such as IL-1, TNFα, IL-6, and INF-y show a circadian pattern, with the highest concentrations at night, suggests a potential role for these molecules in the physiological regulation of sleep in the absence of an immune challenge [28]. In addition, several cytokines and their receptors have been shown to be present and biologically active in the central nervous system of healthy organisms, where they interact physiologically with neuronal circuits associated with sleep regulation (e.g., the serotonergic, GABAergic, and cholinergic systems) [29]. Although the list of cytokines and chemokines that have been shown to affect sleep is extensive, only two, IL-1 and TNFα, have been studied to a sufficient extent to determine their involvement in the regulation of normal physiological sleep in a context other than immune activation [30]. In experimental animals, the administration of IL-1 beta and TNFα promote non-rapid eye movement sleep under physiological and inflammatory conditions [29,31,32].

Alterations in the normal patterns of cytokines that occur during the activated immune response can therefore directly change sleep-wake behavior. This could be the basis for the changes in sleep that occur during infection [33].

According to the above, sleep and inflammation mediators are closely and reciprocally linked. This review will therefore systematically review the published studies assessing the relationship between levels of inflammatory cytokines in saliva and the quality of sleep in a wide perspective including adults and children, and in both healthy subjects and those with a defined disease. Although biomarkers of stress and inflammation have traditionally been studied in blood, salivary cortisol, IL-1β and IL-6 are highly correlated with blood levels [34,35,36]. Saliva can therefore be a diagnostic tool which can be used to monitor sleep in a non-invasive way, without the sampling technique entailing added stress for the patient which implies changes in levels, which could occur as a result of the stress associated with drawing blood. 

In order to analyze the studies published on this topic, we conducted a literature review based on a scoping review design [37,38]. A scoping review has different characteristics from a systematic review, as instead of evaluating the literature to answer a specific question, it systematically analyzes the findings, identifies the most relevant variables and their associations, and determines points for future research. Considering that the area of the salivary biomarkers in sleep research is relatively new, there are no published reviews on this topic to date. The methodology does not differ significantly from a systematic review, although publications are not excluded based on the study design, and no assessment of the quality of each study is necessary, although it is recommended, as in our case. Furthermore, a scoping review is not intended to aggregate results through meta-analysis based on unique outcome, but rather to summarize the most important details to date in order to identify issues, trends, and gaps for future studies. 

## 2. Materials and Methods

### 2.1. Search Process

A search for articles in electronic databases was carried out in October 2020, following the PRISMA criteria [39]. This search resulted in a review that includes a total of 13 published studies. To carry out this search, we formulated the following research question: 

*Scoping Review Question: Is there a Relationship between Sleep Quality and Inflammatory Markers in Saliva?*

The search process was carried out in the following electronic databases: PubMed, EBSCO, and SCOPUS. We initially sought potential primary studies in the databases using the following search string: 

“cytokine” AND “saliva” AND ‘‘sleep’’[Title/Abstract] OR ‘‘interleukin” AND “saliva” AND “sleep” assessment’’[Title/Abstract] OR “inflammatory” AND “biomarkers” AND “saliva” AND “sleep”

This search string was designed after an analysis of the keywords in the relevant literature, which were obtained from several general searches in the resources outlined above. As a result of the search process, 117 studies were identified. Excluding unavailable and duplicated results, we obtained 74 studies.

### 2.2. Inclusion and Exclusion Criteria

The following inclusion and exclusion criteria were defined to address the research question: 

IC1: Articles measuring the relationship between inflammatory cytokines or other inflammatory markers in saliva and the quality of sleep. IC2: Original articles. IC3: Articles written in English or Spanish. EC1: Articles measuring cytokines and other inflammatory biomarkers in blood were excluded.

### 2.3. Studies Selection and Analysis

**Figure 1:**
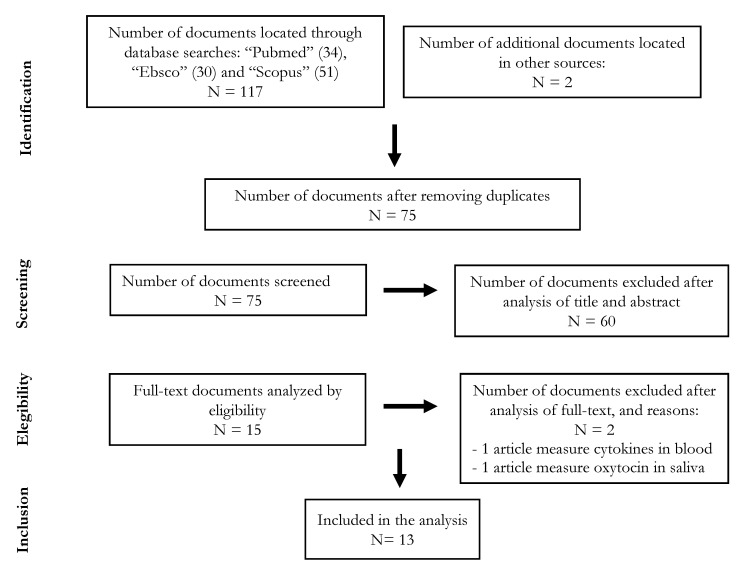
Flowchart according to PRISMA guidelines. (N = number of studies).

Initially, we performed screening on the titles and abstracts to decide whether to include or exclude each study. As a result, from the six sources, a total of 74 studies were selected and 62 were excluded. We read the full text of each primary study included in the preliminary selection in detail to decide whether to include or exclude the study. The primary studies included in the final selection are the relevant papers that comply with the research questions set out in this study. The PRISMA flow chart of the reviewing process is shown in Figure 1.

The participants in the studies selected were healthy adults, shift workers, adults with obstructive sleep apnea syndrome (OSAS) [40,41,42], adults with advanced stage cancer [43], adults with migraine [44], adults with chronic kidney failure (CRF) [45], and children and adolescent populations among which there were people with diagnoses included in the DSM-V [46,47]. The sample characteristics, sleep instruments, primary outcomes related to salivary biomarkers and sleep alterations are shown in Table 1. 

### 2.4. Salivary Inflammatory Markers Measured in the Analyzed Studies

IL-6 was measured in 9 of the 13 articles analyzed [41,43,45,46,47,48,49,50,52]. According to the study published by Li Zhang et al. [49] on nurses who did shift work, IL-6 levels were correlated with the ISI (Insomnia Severity Index). The results published by Nejat Nizam et al. [41] show that TNFα and IL-6 levels were correlated to ISI scores, and salivary concentrations of IL-6 and IL-33 were similar in the two groups of OSA (Obstructive Sleep Apnea Syndrome) and higher than in the control group. According to Pinto et al. [45] the group of patients with chronic kidney failure (CRF) that had poorer sleep quality according to the Pittsburgh Sleep Quality Index (PSQI), also presented higher levels of IL-6 compared to the control group. In the study by El-Sheikh et al. [46], sleep-disordered breathing in children was directly associated with higher levels of salivary IL-6. Reinhardt et al. [52] evaluated sleep quality by means of actigraphy measurements performed in healthy male workers of a welding company who worked morning shifts from 7 a.m. to 5 p.m. Workers with a shorter duration of sleep (<6 h) had higher levels of salivary IL-6 at bedtime and at 2 p.m. than workers with a longer duration of sleep (≥6 h).

In four of the studies analyzed, there is no elevation of daytime IL-6 levels when there is drowsiness, and these effects are maintained or even rise when the quality of sleep improves. According to the studies by Reinhardt et al. [48] on workers who worked shifts, day and night workers produced similar amounts of IL-6. LaVoy et al. [47] recently carried out a study in children/adolescents, in which they found that the concentration of IL-6 in the morning was associated with the prior night’s sleep efficiency and the total amount of minutes spent awake the night after saliva sampling. In the study by Faraut et al. [50], IL-6 values fell significantly after a night with only 2 h of sleep, and this effect normalized after a daytime nap of no more than 30 min at 9:30 a.m. and at 15:30 p.m. Interestingly clinical intervention such as cranial electrotherapy stimulation that are accompanied by an improvement of quality of sleep are also accompanied by corresponding changes in the concentration of IL-6 [43], suggesting it could be a possible marker to monitor the effects of interventions to improve sleep quality.

Seven of the reviewed articles [41,43,44,47,48,49,51] analyze the relationship between IL-1β levels in saliva and sleep. In four of them, no significant changes were detected between the study groups in the levels of IL-1β related to sleep [41,43,48,51]. There was a reduction in this cytokine in only one of the seven articles [49], coinciding with significantly lower insomnia, anxiety, and depression scores in the intervention group. In two others, there was an increase in IL-1β in saliva. One of them was a study focused on the child-adolescent population [47], and in the other [44] the levels of this cytokine were increased, and PSQI values improved after ten weeks of treatment with nVNS.

In the study published by Nizam et al. [41], where they measured sleep with polysomnography and compared IL-1β concentrations between three groups of patients (without OSA, mild/moderate OSA, and severe OSA), the salivary concentrations of IL 1β were similar in all the study groups. This study also measured the levels of IL-21, pentraxin-3 (PTX3), and IL-33. IL-21 and pentraxin-3 (PTX3) concentrations were similar in all study groups. However, the salivary concentrations of IL-33 were similar in the two OSA groups, and were statistically higher than the control group, as was the case with IL-6.

Yennurajlingam et al. [43] measured the levels of IL-1β in saliva in individuals with advanced cancer. These people had at least one symptom of moderate intensity of depression, anxiety, sleep disorders, and pain, and received stimulation by cranial electrotherapy for 4 weeks, with no significant changes in their IL-1β levels. In the study published by Boström et al. (2019) [44], IL-1β levels in the saliva of migraine patients receiving treatment for 10 weeks increased after cervical non-invasive vagus nerve stimulation (nVNS) therapy for 10 weeks, and the secondary endpoints did not change. In another study published by Reinhardt et al. [48], using a sample of 38 healthy shift workers, IL-1β levels were similar for both day and night shift workers. Both groups presented a significant daily variation in their pattern of IL-1β. However, the pattern of daily variation observed among day workers, peaking after awakening, was not observed in night shift workers. Night shift workers showed partially adjusted daily variation patterns for salivary IL-1β.

In contrast to all these results, a recent study published by Zhang et al. [49] found that salivary TNFα and IL-6 levels were directly correlated with insomnia severity in nurses who worked shifts and the administration of Shimian granules (a traditional Chinese herbal medicine) improve sleep and decreased the level of these inflammatory markers in saliva suggesting that similar to other intervention mentioned before [43], they could be a possible marker to monitor the effects of interventions to improve sleep quality. 

In a study by Reinhardt et al.’s group [51] and carried out healthy workers who worked shifts and in whom sleep was measured with actigraphy for 10 consecutive days, no relationship was found between levels of salivary IL-1ß and quality of sleep. In this study, IL-1ß levels were higher upon awakening than at bedtime for all the workers on the last working day (Friday) regardless of their work shift, bedtime, sleep efficiency, or total duration of the sleep. In the infant-adolescent population, we found only one study published by LaVoy et al. [47] hat measured salivary IL-1β concentration and found higher levels of IL-1β in the morning associated with a better quality of sleep measured the night before.

Evidence regarding the levels of TNFα in saliva related to sleep is reported in three articles, with different results in terms of the associations with sleep parameters [45,48,49]. In one of them, the salivary TNFα levels increase, in another they decrease, and in the third they are unchanged. Zhang et al. (2020) [49] concluded that the biochemical indices of TNFα in saliva fell significantly when the participants were treated with the traditional herbal medicine Shimian granules, and improved sleep, as did the indices of IL-1β and IL-6. 

In contrast, in the study by Pinto et al. [45] demonstrated that in patients with chronic kidney failure undergoing hemodialysis higher score on the global PSQI, a lower quality of sleep and higher prevalence of sleep disorders are associated with higher levels of TNF-α. In contrast, TNF-α levels were similar for day and night shift workers, with higher daily production upon waking, in the morning for day workers and in the afternoon for night workers [48].

The enzyme prostaglandin-endoperoxide synthase 2 (PTGS2) (called also COX-2) catalyzes the conversion of arachidonic acid to prostaglandins in two steps. First, hydrogen is abstracted from carbon 13 of arachidonic acid, and then two molecules of oxygen are added by the PTGS2 (COX-2), giving PGG2. Second, PGG2 is reduced to PGH2 in the peroxidase active site. The synthesized PGH2 is converted to prostaglandins (PGD2, PGE2, PGF2α), prostacyclin (PGI2), or thromboxane A2 by tissue-specific isomerases which all increased during inflammatory states. The transcription of PTGS2 gene was measured in saliva in one study [42], showing that the inflammatory transcripts of several prostaglandins are elevated in conditions of excessive daytime sleepiness.

Finally, salivary myeloperoxidase (MPO) was measured in one article [40]. MPO is a peroxidase enzyme that is released from the azurophilic granules of polymorphonuclear leukocytes during the inflammatory process and has been shown to be a sensitive predictor of inflammation. Salivary MPO levels were significantly higher in the OSA group compared to healthy controls. This indicates that higher levels of MPO in saliva may be a marker of persistent local oropharyngeal inflammation in subjects with OSA.

The techniques used to analyze the inflammatory markers in saliva were the Enzyme-Linked Immuno Sorbent Assay (ELISA) for inflammatory cytokines [41,43,44,46,47,48,49,50,51]. In contrast, myeloperoxidades were measured using flux cytometry [40], and RNA transcripts for PTGS2 were analyzed using real-time PCR [42].

### 2.5. Sleep Detection Methods Used the Analyzed Studies

Sleep detection methods can be classified into two large groups according to whether or not they need medical assistance [53]. Polysomnography (PSG) [54,55,56] is a medical procedure composed of several concurrent but independent tests that monitor different bodily functions during sleep, which are recorded for further study using different channels. PSG was used in 6 of the 13 articles analyzed in this review [40,42,47,48,51,52]. This method is the most advanced tool for diagnosing many sleep disorders. It was used to calculate the apnea-hypopnea index in three of the analyzed studies [40,41,42]. Sleep questionnaires are a very inexpensive and rapid test and analyze the patient’s (subjective) self-perception about quality of sleep in a quantitative way. However, their subjectivity does not necessarily render these questionnaires inaccurate, as demonstrated by several validation studies [57,58,59,60,61,62]. In the studies analyzed, the following sleep questionnaires were used:

- The Pittsburgh Sleep Quality Index (PSQI) [63]: used to record the sleep quality and patterns of sleep in adults. It contains 9 items (4-point scale). This questionnaire was used in 4 of the studies analyzed in this review [43,44,45,49].
- The Insomnia Severity Index (ISI) [64]: records the nature, severity, and impact of insomnia and treatment response in adults. It contains 7 items (5-point scale). This questionnaire was used in only one [49] of the studies analyzed.
- The Epworth Sleepiness Scale (ESS) [65]: measures the level of daytime sleepiness, and the average propensity to sleep in daily life. It contains 8 items (4-point scale). This questionnaire was used in only one [42] of the analyzed studies.
- The Children’s Sleep Habits Questionnaire (CSHQ) [66]: designed to examine sleep behavior in young children. It contains 45 items (three-point scale). It is filled in by the parents and was used in one [46] of the analyzed studies.
- The 10-item sleep-wake problem subscale of the School Sleep Habits Survey (SSHS) assesses the frequency with which sleep is interrupted, ranging from 1 (never) to 5 (every day). Higher scores on this instrument indicate greater sleep disturbance. The SSHS has shown strong psychometric properties [67] and was used in one [46] of the articles analyzed in this review. Sleep diaries represent another subjective measurement that is completed over a longer period of time (typically several times during one to two weeks) and were used in two studies including in the review [47,52].

Contact hardware devices to assess sleep are small devices that can be attached to the wrist, chest, ankle, or head. Some of these devices use the Cartesian system to record the activity of the body, and they are therefore known as actigraphs. Modern actigraphs collect information from various sensors (accelerometer, gyroscope, thermometer, etc.). The information collected is then combined and processed by specialized algorithms [68,69] to detect sleep. Five of the articles analyzed used actigraphy to detect the sleep [46,47,48,51,52]. The activity records were collected for one week [46] and ten days [48].

### 2.6. Evaluation of the Quality of the Methodology 

The Agency for Healthcare Research and Quality (AHRQ) checklist was used to assess the quality of the included studies. We used the checklist for Cross-Sectional/Prevalence Studies since all the studies included in the scoping review used these study designs (refs. Website: http://www.ncbi.nlm.nih.gov/books/NBK35156/).

The AHRQ checklist is shown in Table 2. The first 8 items on the checklist were mostly analyzed in the studies. The studies by Reinhardt et al. [48] and LaVoy et al. [47] have the highest methodology clarity (fulfilling 7 out of 8 items) based on the checklist items, and many others fulfilled 8 out of 8 items [43,45,46,49,50]. Due to the type of the design of the studies, none of them explained how missing data were handled in the analysis (item 9) or clearly summarized the patients’ response rates and the completeness of data collection (item 10). Only one study [51] evaluated the follow-up and the percentage of patients for which incomplete data or follow-up was obtained (item 11).

1. Define the source of information (survey, record review).
2. List inclusion and exclusion criteria for exposed and unexposed subjects (cases and controls) or refer to previous publications.
3. Indicate time period used for identifying patients.
4. Indicate whether or not subjects were consecutive if not population-based.
5. Indicate if evaluators of subjective components of study were masked to other aspects of the status of the participants.
6. Describe any assessments undertaken for quality assurance purposes (e.g., test/retest of primary outcome measurements).
7. Explain any patient exclusions from analysis.
8. Describe how confounding was assessed and/or controlled.
9. If applicable, explain how missing data were handled in the analysis.
10. Summarize patient response rates and completeness of data collection.
11. Clarify what follow-up, if any, was expected and the percentage of patients for which incomplete data or follow-up was obtained.

The main strength and limitations of the analyzed studies are summarised in Table 3.

## 3. Discussion

Reduced sleep has adverse effects on health: the metabolism is altered and there is an increase in the secretion of C-reactive protein, interleukin IL-6, and TNFα [70]. This study has reviewed various scientific publications on the relationship between inflammatory cytokines detected in the saliva during wakefulness in the adult population and in the infant-adolescent population and the quality of sleep.

Sleep is known to influence cytokine levels and in particular, IL-6 is secreted subject to circadian rhythms, and increases with the feeling of drowsiness [71]. The lack of sleep and sleep disturbance have been shown to increase daytime plasma IL-6 levels in adults [72]. However, most studies linking IL-6 to sleep have measured IL-6 in blood rather than saliva.

This review includes 13 articles that measure salivary cytokine levels. Nine of the studies reviewed looked at IL-6 levels in saliva. A greater elevation of IL-6 levels during the day is demonstrated in patients suffering from disorders with excessive daytime sleepiness in five of them [41,45,46,49,52]. This supports the initial hypothesis that relates the association between lack of sleep and increased levels of IL-6. However, contrary to the initial hypothesis, in children and adolescents, salivary cytokine levels were higher in those who experienced better sleep [47]. These results show that it is necessary to continue this research in order to determine the sensitivity of salivary IL-6 to differences and alterations in the duration and quality of sleep, especially in children and adolescents, since this study indicates that children with longer and more efficient sleep have higher levels of pro-inflammatory mediators in saliva upon waking. It is necessary to determine the relationships between sleep and IL-6, because this is a critical signaling molecule in the immune response to pathogens, and chronically elevated levels are related to the onset of cardiovascular, metabolic and autoimmune diseases [73,74,75]. Among the salivary biomarkers analyzed in the study by LaVoy et al. [47], IL-1β was positively correlated with sAA (salivary α-amylase) and IL-6, and negatively correlated with cortisol. SE (sleep efficiency) was significantly associated with IL-6 and accounted for 15% of the unique variance, while a higher SE was associated with higher levels of IL-6. TST was significantly associated with IL-1β and SOL was negatively associated with IL-1β, indicating that a shorter SOL was associated with higher IL-1β. Higher levels of morning stress and inflammatory biomarkers were associated with poorer sleep the following night. In contrast to the findings for the night before, IL-1β was positively correlated with SOL (sleep onset latency) the following night. In other words, IL-1β levels were associated with a longer SOL.

Like IL-6, IL-1β also plays a key role in promoting sleep [76]. Sleep deprivation has been related to an increase in IL-1β in stimulated cells [77] and there is evidence that there is an increase in markers of systemic inflammation such as IL-1β when partial sleep deprivation is repeated for several days [19,78]. Although blood IL-1β and sleep have been studied in animals and in experimental settings, few studies have examined whether salivary IL-1β values influence sleep quality.

Seven of the articles analyzed in this review highlight the relationship evidenced between levels of IL-1 β in saliva and sleep. In general, IL-1ß production is not associated with sleep disturbances, as reported in the literature [77,79]. Only one article specified that IL-1ß values could be affected by night work, and there was an increase in this cytokine in the measurement carried out on the last day of work regardless of the work shift, bedtime, sleep efficiency or total sleep duration [51]. It is important to note that this was a study with a very small sample size (N = 5), so it is possible that no association between cytokines and sleep markers could be observed due to the reduced size of the sample. In four other studies, there were no significant changes between the study groups in the levels of IL-1β related to sleep, and in one study there was an increase in IL-1β with the improvement of the quality of sleep of the child-adolescent population studied [47]. These analyzed data contradict the initial assumption, which related sleep deprivation with an increase in IL-1β in stimulated cells [77]. Further analysis is needed to understand the effects of sleep on salivary IL-1ß production.

Three of the thirteen articles analyzed contain evidence for levels of TNF-α in saliva related to sleep, with very different results. In one of them the TNF-α values increase, in another they decrease, and in the third there are no variations. These results contradict the initial hypothesis which related a reduction in sleep with an increase in tumor necrosis factor alpha concentration. However, TNF-α levels can affect sleep quality through its regulatory effects on pineal melatonin synthesis, a hormone associated with sleep initiation and maintenance. In fact, it has been suggested that increased circulating TNF-α, through NFκB activation, leads to a blockade of nocturnal melatonin production induced by sympathetic stimulation of pinealocytes [80]. Likewise, it is well documented that melatonin can block transcriptional factors that induce pro-inflammatory cytokines, among others, TNF-α [81]. This negative relationship between TNF- α and melatonin levels in saliva and its association with sleep has been observed [82], in patients with chronic renal failure. Similarly, it has been suggested, in a sample of nurses with clinical insomnia working shifts, that Shimian Granules increased sleep quality by enhancing in opposite directions the salivary levels of melatonin and TNF-α [49]. 

The levels of melatonin, TNFα, and IL-6 were correlated with the Insomnia Severity Index [49] so that the higher the insomnia score, the higher the levels of TNF-α and IL-6 and the lower the melatonin levels. The results of a study by Reinhardt et al. in 2012 suggest that the variation pattern of salivary IL-1ß may be altered by night shift work and that the alteration of melatonin rhythms seems to occur simultaneously with alterations in IL-1ß levels in saliva when wake up and at bedtime. These results are consistent with other studies [83] which conclude that the alteration of the circadian system directly affects the production of inflammatory cytokines. However, because the sample consisted of only five participants, it was not possible to establish a clear association between melatonin and IL-1ß. In another study also conducted by Reinhardt et al. in 2018 [48], this time with a larger sample, low salivary melatonin concentrations were detected in night shift workers and a lack of daily melatonin rhythm, which could be caused by a chronic circadian alteration. Likewise, no clear pattern of daily IL-6 variation was observed among night workers, and daily IL-1β salivary variation patterns for night shift workers showed partially adjusted patterns. This absence of daily variation of IL-6 would reinforce the hypothesis that the daily rhythm of this cytokine is determined by the circadian system and would justify the non-elevation of IL-6 levels in people who have it altered, such as workers on a night shift.

The role of salivary cytokines and melatonin as biomarkers to assess sleep disturbances needs to be further investigated in the context of the emerging evidence about the immune-pineal gland axis interplay.

In two of the articles studied, other inflammatory markers were measured in saliva in a population diagnosed with obstructive sleep apnea. The transcription factors ANXA1, β2M, PTGS2 and CASP1 [42] and myeloperoxidase (MPO) [40] were measured. High values of the markers were associated with alterations in sleep quality in both studies. Only one study [40], evaluated the level of inflammatory markers in both saliva and blood in patients with obstructive sleep apnea (OSA), and observed an increase in the concentrations of MPO (myeloperoxidase) in both serum and saliva compared to healthy controls although such increase was greater in the saliva samples. The authors argue that such increase in the saliva samples could likely be due to differences in the course and intensity of oropharyngeal and systemic inflammation. Oropharyngeal inflammation may appear as a local and acute high-grade response that precedes systemic inflammation, while in the systemic, the response may be low-grade and less prominent [40,84].

More research is needed due to the fact that very few studies have examined salivary cytokines in relation to sleep. Furthermore, taking into account such disparate results of IL-6 according to the population sample of the studies examined, more studies in children and adolescents would have to be carried out. The activation of the immune system resulted in an increase in cytokines upon awakening and a longer duration and efficiency of sleep in the study participants [47]. Due to paucity of data on salivary biomarkers and the diverse outcomes related to sleep investigated so far e.g., subjective/objective sleep quality, sleep onset time, daytime sleepiness, etc., as well as the heterogeneity of different studied populations (healthy control subjects, works shifts, or individuals with a definite disease) is still difficult to make recommendations about a salivary markers panel useful in clinical practice. Among the identified salivary markers is crucial in future studies to analyze their ability in predicting both subjective or objective sleep quality assessments and to evaluate their changes after interventions that improve sleep quality or sleep-related disorders.

More research is therefore needed to determine the sensitivity of salivary inflammatory markers in the general population and in children and adolescents as salivary markers of sleep quality but useful some inflammatory markers emerged from this literature review.

## 4. Conclusions

Sleep influences cytokine levels and its alteration is associated with adverse effects on metabolism and increased levels of pro-inflammatory molecules. For this reason, cytokines measured in saliva can be used as biomarkers of sleep quality. However, the current scientific literature related to the measurement of cytokines in saliva to objectively assess sleep is scarce, and only 13 scientific articles that deal with this topic have been found. Reduced or fragmented sleep increases the secretion different inflammatory markers of C-reactive protein, IL-6, and TNFα [70]. In this review, we found a quite nice consensus among the studies that associate poor sleep quality with altered levels of salivary IL-6 and supported by five of the nine studies analyzed which measured this cytokine in saliva, in which there is elevation of IL-6 levels during the day in patients suffering from sleep alterations [41,45,46,49,52]. However, in four of the studies analyzed, the elevation of daytime IL-6 levels does not occur when there is drowsiness, and these effects are maintained or even rise when the quality of sleep improves. Only three articles evaluate TNFα and no summary conclusion can be done regarding this cytokine in saliva because of very different results reported with TNFα values increased [45], reduced [49], and unchanged [48]. Seven of the articles reviewed analyzed the relationship between IL-1β levels in saliva and sleep quality, and no significant associations were found in four of them [41,43,48,51]. In one of the seven articles, there was a reduction of IL-1β [49], and an increase in IL-1β in saliva in the other two [44,47]. Emerging salivary inflammatory markers such as IL-33 and the inflammatory inducible COX-2 enzyme [42] or myeloperoxidase [40] deserves further studies since it has been associated with sleep quality in recent studies. However, further studies assessing whether inflammatory markers in saliva can represent useful a tool to diagnose and monitor the effects of pharmacological and non-pharmacological interventions aimed to improve sleep quality. The comparison of measurements between saliva and blood samples is particularly warranted in order to assess the salivary levels of these markers as a surrogate of systemic levels. The role of nutritional status, inflammatory diseases and drugs on salivary inflammatory markers also need a careful analysis in order to shed new lights on possible confounding factors in its determination and data interpretation. 

## Figures and Tables

**Table 1:** Main characteristics of the studies included in the scoping review.

Reference	N/Sex	Mean Age	Sample Characteristics	Instrument(s) Used to Assess Sleep Quality	Measurement	Relationship Between Salivary Inflammatory Markers and Sleep	Study Design
Reinhardt et al. 2019 [48]	38 males	Day workers 32.1 years and night workers 32.5 years	Healthy controls (workers with no shifts but one group has a permanent day shift, from 7:00 to 17:00, and the other group has a permanent night shift, from 21:00 to 6:00).	Actigraphy	Sleep disturbance. Circadian disturbance	The levels of TNFα and IL-1β were similar for day and night shift workers, with higher daily production upon awakening, for day workers, for evening workers and for night workers. Day and night workers produced similar amounts of IL-6.	Observational Prospective
Zhang et al., 2020 [49]	38 females	29.0 years	Healthy controls (nurses with shifts)	Insomnia Severity Index (ISI) Pittsburgh Sleep Quality Index (PSQI)	Subjective insomnia severity Subjective quality of sleep	The levels of TNFα, IL-1β and IL-6 in saliva were lower with the Shimian granules (traditional Chinese herbal treatment for sleep disorders) treatment. TNFα and IL-6 levels were directly correlated with insomnia severity.	Experimental Clinical trial (placebo-controlled)
Faraut et al., 2015 [50]	11 males	27.0 years	Healthy controls (non-smokers)	Polysomnography	Monitor sleep-wake cycle after sleep restriction	IL-6 levels were lower at 10:00 a.m. and 7:00 p.m. on days with restricted nighttime sleep. The decrease in IL-6 normalized after a daytime nap (<30 min)	Experimental
Reinhardt et. al. 2012 [51]	5 males	Not specified	Healthy controls (workers with shifts: 3 night (21:00–6:00) and 2 day (7:00–17:00))	Actigraphy	Sleep deprivation caused by shift work	IL-1ß levels were higher upon awakening than at bedtime for all workers regardless of work shift, bedtime, sleep efficiency, or total sleep duration.	Observational Prospective
Reinhardt et al., 2016 [52]	21 males	32.1 years	Healthy controls (workers with no shifts)	Actigraphy	Sleep duration	Workers with a duration of sleep <6 h had higher levels of salivary IL-6 at bedtime IL-6 and at 14:00 h compared to workers with a longer duration of sleep (≥6 h). Workers with longer sleep duration presented a lower production of salivary IL-6 around 14:00 h, during work shift, and a higher production at awakening and at bedtime	Observational
Nizam et al., 2014 [41]	52 (32 males;20 females)	46.6 years	Healthy controls Individuals with sleep apnea (OSA)	Polysomnography Apnea-hypopnea index	Presence and severity of OSA	Salivary concentrations of IL-6 and IL-33 were statistically higher in OSA group compared to the control group. The concentrations of IL-1β and IL-21 were similar in all groups.	Observational Prospective
Yennurajalingam et al., 2017 [43]	52 (20 males; 32 females)	59 years (median)	Patients with advanced cancer	Pittsburgh Sleep Quality Index (PSQI)	Subjective quality of sleep	Improvement in the total PSQI sleep score by 2 points, and a significant improvement in the daytime PSQI dysfunction after cranial electrotherapy stimulation (CES). A trend towards improvement in IL-6 (but not in IL-1beta) after CES.	Experimental One group open label pre- and post-intervention
Boström et al., 2019 [44]	24 females	47.6 years	Healthy controls and Individuals with migraine	Pittsburgh Sleep Quality Index (PSQI)	Subjective quality of sleep	Migraine-related abnormalities in sleep architecture improved after ten weeks of vagus nerve stimulation (nVNS) treatment. IL-1β levels in saliva increased after nVNS therapy, producing values 2.5 times higher than those measured in healthy controls.	Observational Prospective Case-control study
Pinto et al. 2015 [45]	39 (sex not specified)	54.9 years	Healthy controls and patients with chronic renal failure (CRF).	Pittsburgh Sleep Quality Index (PSQI)	Subjective quality of sleep	The CRF group obtained a higher score than the control group on the global PSQI, i.e., lower sleep quality and a higher prevalence of sleep disorders. The CRF group also presented higher TNFα and IL-6 contents than the control group.	Experimental Clinical cross-sectional
El-Sheikh et al. 2007 [46]	64 children (28 males; 36 females)	8.8 years	Healthy controls	Actigraphy School Sleep habits Survey using Sleepiness and Morning/Eveningness (to children) Children’s Sleep Habits Questionnaire (CSHQ) (to Parents)	Quality and quantity of sleep, sleep onset time, daytime sleepiness	Children with higher salivary IL-6 levels reported increased Eveningness predispositions and their parents reported higher levels of sleep disordered breathing.	Observational Cross-sectional and Prospective
Thimgan et al., 2015 [42]	40 (22 males; 18 females)	Control group: 40.8 years Sleep apnea (OSA) group:49.0 years	Healthy controls and individuals with sleep apnea	Polysomnography Apnea-hypopnea index Epworth Sleepiness Scale	Severity of OSA	The transcripts of the enzyme prostaglandin-endoperoxide synthase 2 (PTGS2) in saliva was increased in patients with sleep apnea.	Observational Prospective
Akpinar et al., 2012 [40]	56 (40 males; 16 females)	Control group: 44.7 years; OSA group: 43.8 years	Healthy controls and individuals with obstructive sleep apnea (OSA)	PolysomnographyApnea-hypopnea index (AHI)	Severity of OSA	Salivary myeloperoxidase (MPO) levels were significantly higher in the OSA group compared to controls and the increase in MPO in saliva correlated with the severity of OSA according to the AHI and oxygen desaturation index.	Observational Prospective
LaVoy et al. 2020 [47]	55 children (26 males; 29 females)	12.2 years	Healthy controls and children with different type of psychiatric disorders	Actigraphy 10-item sleep-wake problems subscale of the School Sleep Habits Survey (SSHS).	Total sleep time, sleep efficiency, number of waking episodes, number of minutes spent awake during the night, sleep onset latency	Salivary Il-1β was positively associated with prior night total sleep time and negatively associated with sleep onset latency, and IL-6 was associated with greater sleep efficiency. Higher IL-1β predicted a longer sleep onset latency the following night.	Observational Prospective

**Table 2:** The Agency for Healthcare Research and Quality (AHRQ) checklist was used to assess quality of the included studies.

Article\Items	1	2	3	4	5	6	7	8	9	10	11
El-Sheikh et al., 2007 [46]	Y	Y	Y	Y	U	Y	N	Y	NA	U	U
Reinhardt et al., 2012 [51]	Y	U	Y	Y	U	Y	Y	U	U	U	U
Akpinar et al., 2012 [40]	Y	Y	Y	Y	U	N	N	Y	NA	U	U
Nizam et al., 2014 [41]	Y	Y	Y	Y	U	N	N	N	U	U	U
Pinto et al., 2015 [45]	Y	Y	U	Y	U	Y	Y	Y	U	U	U
Faraut et al., 2015 [50]	Y	Y	Y	Y	Y	N	N	Y	U	U	U
Thimgan et al., 2015 [42]	Y	N	U	Y	N	Y	N	Y	NA	U	U
Reinhardt et al., 2016 [52]	Y	N	U	U	N	Y	N	N	U	U	U
Yennurajalingam et al., 2017 [43]	Y	Y	Y	Y	N	Y	Y	U	U	U	Y
Reinhardt et al., 2019 [48]	Y	Y	Y	Y	Y	Y	Y	U	U	U	U
Boström et al., 2019 [44]	Y	Y	Y	Y	N	N	Y	U	U	U	U
Zhang et al., 2020 [49]	Y	Y	Y	Y	Y	N	Y	U	U	U	U
LaVoy et al., 2020 [47]	Y	Y	Y	Y	Y	Y	Y	U	U	U	U

Y: yes; N: No; U: Unknown; NA: not assessed.

**Table 3:** Main strengths and limitations of the studies included in the scoping review.

Reference	Strengths	Limitations
El-Sheik et al., 2007 [46]	Homogeneous sample: healthy children (age = 8–9, similar gender proportion) Sample size: N = 64 Sample collected at two different times (3 p.m. and 5 p.m.) Combines objective and subjective sleep assessment methods: one-week actigraphy + School Sleep habits Survey (children) and Children’s Sleep Habits Questionnaire (CSHQ) (parents)	Only IL-6 was studied
Reinhardt et. al., 2012 [51]	Homogeneous sample: healthy men Daytime workers and overnight workers were compared. Sample collected at two different times: wake time and midnight. Objective and subjective methods combined: Diaries + actigraphy Includes the study of melatonin in saliva	Small sample: N = 5 No analysis with the secondary variables assessed (depression, anxiety)
Akpinar et al., 2012 [40]	Narrow age range (control: 44.7 ± 13.75; experimental: 43.79 ± 12.72) Sample size N = 56 Healthy people and people with OSA are studied Objective and subjective methods combined: Polysomnography and Epworth Sleepiness Scale (ESS) Mieloperoxidase (MPO) was measured on blood and salivaIMC was measured as a secondary variable	Only MPO and PCR levels were assessed (other inflammatory markers such cytokines were not assessed).
Nizam et al., 2014 [41]	Polysomnography is applied to all participants (N = 52) Healthy people and people with OSA are studied Several cytokines were measured: IL-6, IL-33, IL-1β, IL-21, and pentraxin-3 (PTX3)	Unbalanced sample: 32 men and 20 women Wide age range: 21–64 years Only one sample collected
Faraut et al., 2015 [50]	Homogeneous sample: healthy men (age = 25–32) Polysomnography Sample collected at several time points (every two hours)	Small sample: N = 11 Only IL-6 was assessed
Pinto et al., 2016 [45]	Sample collected at several times (every four hours) Includes the study of melatonin in saliva	Heterogeneous sample: healthy people and people with chronic renal failure. Wide age range: 29–79 years (N = 39) Only TNFα and IL-6 were assessed Samples were collected along a period of only 24 h. Cytokines were assessed only in blood (not in saliva) Sleep assessed with only one questionary: Sleep Quality e Index Pittsburgh (PSQI)
Thimgam et al., 2015 [42]	Objective and subjective methods combined: Polysomnography and Epworth Sleepiness Scale (ESS) A polysomnogram was produced for all patients (N = 40) IMC was measured as a secondary variable	Heterogeneous sample: healthy people (5 men/3 women), OSA patients (11 men/3 women), and patients with suspected OSA (6 men/12 women) Inflammatory transcriptions were assessed, but not cytokines
Reinhardt et al., 2016 [38]	Homogeneous sample: healthy men workers with day work shift. Narrow age range (32.14 ± 7.61) Objective and subjective methods combined: Actigraphy and diaries Sample collected at three different times (wake, 2pm and bedtime)	Small sample: N = 21 Only IL-6 was studied
Yennurajalingam et al., 2017 [43]	Sample (N = 52) collected at two different times (wake and bedtime) IL-1β and IL-6 were assessed Secondary variables were measured: anxiety, depression with HADS (Edmonton Symptom Assessment Scale), pain with BPI (Brief Pain Inventory) and drugs used.	Heterogeneous sample: advanced cancer patients with at least one symptom of depression, anxiety, sleep disorders, and pain. Unbalanced sample: 32 men and 20 women Sleep assessed with only one questionary: Pittsburgh Sleep Quality Index (PSQI) Sample collected only once a week
Reinhardt et al. 2019 [48]	Homogeneous sample: healthy men workers. Narrow age range: day workers 32.14 ± 7.61 years old and night workers 32.59 ± 5.62 years old Different work shifts were analyzed Actigraphy used for 10 days (24 h/day) Sample collected at two different times TNFα, interleukin-1β (IL-1β), and IL-6 were studied Melatonin levels in saliva were assessed.	Sample size: N = 38 Different number of workers in each group (day: 21; night: 17) No secondary variable was studied
Boström et al., 2019 [44]	All participants were women. All samples collected at the same time Secondary variables were studied: functional capacity with MIDAS (Migraine Disability Assessment), depressive symptoms with BDI (Beck Depression Inventory) and quality of life (EuroQuol EQ-5D-5L)	Heterogeneous simple (N = 24): healthy women and women with migraine Wide age range: 34–65 years Sleep assessed with only one questionary: Pittsburgh Sleep Quality Index (PSQI) Samples were collected only twice along the whole research (at the beginning and at the end, after 10 weeks with nVNS). Only IL-1β was studied
Zhang et al., 2020 [49]	Homogeneous sample: nurse women that work in rotative work shifts. Daily samples collected Different cytokines, e.g., IL-6, IL-1β, and TNFα were studied. Melatonin levels in saliva were assessed. Secondary variables related to sleep were studied: insomnia, anxiety, depression (with HADS) and alert level (with PVT, psychomotor vigilance task)	Sample size: N = 32 Different sample size for the control (13) and experimental groups (19) Sleep assessed with only subjective methods: Pittsburgh Sleep Quality Index (PSQI) and Insomnia Severity Index (ISI) Samples were collected only once a week
LaVoy et al., 2020 [47]	Similar gender proportion (53% women) Narrow age range: 8–16 years Objective and subjective methods combined: Actigraphy, sleep diaries and the School Sleep Habits Survey (SSHS) Daily simple collection (at wake time) All samples were analyzed twice IL-6 and IL-1β were studied Secondary variables were studied: the z-score of body mass index (zBMI), collection time, and season of data collection.	Heterogeneous sample: healthy people and people with at least a diagnostic of attention deficit hyperactivity disorder (*n* = 1), generalized anxiety disorder (*n* = 5), major depressive disorder (*n* = 2), social anxiety disorder (*n* = 4), specific phobia (*n* = 3), pervasive developmental disorder (*n* = 1), depressive disorder not otherwise specified (*n* = 1), and disruptive mood dysregulation disorder (*n* = 1).
